# Targeted screening for third-generation cephalosporin-resistant *Enterobacteriaceae* carriage among patients admitted to intensive care units: a quasi-experimental study

**DOI:** 10.1186/s13054-015-0754-7

**Published:** 2015-02-10

**Authors:** Cédric Dananché, Thomas Bénet, Bernard Allaouchiche, Romain Hernu, Laurent Argaud, Olivier Dauwalder, François Vandenesch, Philippe Vanhems

**Affiliations:** Infection Control and Epidemiology Unit, Edouard Herriot Hospital, Hospices Civils de Lyon, 5, place d’Arsonval, 69437 Lyon, Cedex 03 France; Epidemiology and Public Health Group, University of Lyon 1, 8, avenue Rockefeller, 69373 Lyon, Cedex 08 France; Intensive Care Unit, Edouard Herriot Hospital, Hospices Civils de Lyon, 5, place d’Arsonval, 69437 Lyon, Cedex 03 France; Medical Intensive Care Unit, Edouard Herriot Hospital, Hospices Civils de Lyon, 5, place d’Arsonval, 69437 Lyon, Cedex 03 France; Institut of Microbiology, Department of Bacteriology, East Hospital Complex, Hospices Civils de Lyon, 59 boulevard Pinel, 69677 Bron, France

## Abstract

**Introduction:**

Identification of third-generation, cephalosporin-resistant *Enterobacteriaceae* (3GC-RE) carriers by rectal screening at admission seems to be an important step in the prevention of transmission and outbreaks; however, little is known about its effectiveness. The aim of this study was to evaluate the impact of ‘targeted screening’ at patient admission to intensive care units (ICUs) on the incidence of 3GC-RE hospital-acquired infections (HAIs) and compare it to ‘universal screening’.

**Methods:**

We undertook a quasi-experimental study of two ICUs (unit A: intervention group; unit B: control group) at a university-affiliated hospital between 1 January 2008 and 31 December 2011. In unit A, patients were screened universally for 3GC-RE at admission during period 1 (1 January 2008 through 30 September 2010). During period 2 (2011 calendar year), the intervention was implemented in unit A; patients transferred from another unit or hospital were screened selectively. In unit B, all patients were screened throughout periods 1 and 2. 3GC-RE-related HAI incidence rates were expressed per 1,000 patient-days. Incidence rate ratios (IRRs) were examined by multivariate Poisson regression modelling.

**Results:**

In unit A, 3GC-RE-related HAI incidence rates decreased from 5.4 (95% confidence interval (CI), 4.1 to 7.0) during period 1 to 1.3 (95% CI, 0.5 to 2.9) during period 2 (*P* < 0.001). No changes were observed in unit B between periods 1 and 2 (*P* = 0.5). In unit A, the adjusted incidence of 3GC-RE-related HAIs decreased in period 2 compared with period 1 (adjusted IRR, 0.3; 95% CI, 0.1 to 0.9; *P* = 0.03) independently of temporal trend, trauma and age. No changes were seen in unit B (*P* = 0.4). The total number of rectal swabs taken showed an 85% decrease in unit A between period 1 and 2 (*P* < 0.001).

**Conclusions:**

Targeted screening of 3GC-RE carriers at ICU admission was not associated with an increase in 3GC-RE-related HAI incidence compared with universal screening. Total number of rectal swabs decreased significantly. These findings suggest that targeted screening may be worth assessing as an alternative to universal screening.

## Introduction

Third-generation cephalosporin-resistant *Enterobacteriaceae* (3GC-RE), and particularly extended-spectrum β-lactamase-producing *Enterobacteriaceae* (ESBL-E), have become a global concern since the beginning of the 21st century [1]. The incidence of ESBL-E has increased in both community and hospital settings [2]. The European Antimicrobial Surveillance Network has reported major increases of third-generation cephalosporin-resistant *Escherichia coli* proportions in health care institutions, ranging from 1.7% in 2002 to 8% in 2009 [3]. Similar trends have been noted with ESBL-E infections by the French National Surveillance Network for Multidrug-Resistant Bacteria (BMR-RAISIN). The overall incidence rate increased between 2002 and 2012 from 0.13 to 0.53 per 1,000 patient-days, and in intensive care units (ICUs) the incidence rate during the same period increased from 0.79 to 2.36 per 1,000 patient-days [4], a rate three- to fivefold higher in ICUs than in non-ICU surgical or medical departments [4,5]. Control of ESBL-E spread is therefore urgently required [6].

Many infection control measures, such as contact isolation of patients with colonization or infection, antimicrobial stewardship programmes, active screening culture (ASC) and selective digestive decontamination (SDD), have been proposed to control bacterial transmission [7]. According to a report of a French study of three ICUs, an ESBL-E outbreak was controlled by the association of ASC for all patients at admission with contact isolation [8]. Without evidence supporting specific infection control strategies, no definite guidelines can be implemented in nonoutbreak settings [9]. Only general recommendations on the control of Gram-negative organisms are currently available [10,11]. A 2011 literature review revealed a dearth of research on the control of ESBL-E transmission in hospitals [12]. The lack of evidence-based guidelines, with disparities in infection control practices, may be factors behind the increase in ESBL-E incidence [13].

The importance of ASC is unclear in the absence of outbreaks [14]. ‘Universal screening’ is costly and may not be needed when patient-to-patient transmission [15] and prevalence of carriage at patient admission are low [16]. An option is ‘targeted surveillance’ of patients with severe underlying disease or risk factors for ESBL-E carriage at admission [17,18]. Recent studies have implicated several risk factors for ESBL-E colonization at admission: prior ESBL-E carriage, transfer from hospitalization units and especially long-term care facilities or ICUs, coming from a high-prevalence country, poor functional status, current antibiotic use and chronic renal insufficiency [14,17,19-23].

In our institution, universal screening is performed at ICU admission. In 2010, targeted screening at ICU admission for patients transferred from another unit or hospital was implemented in one of the ICUs because of high costs and resource constraints. We hypothesized that this targeted screening at admission does not increase 3GC-RE-related hospital-acquired infection (HAI) incidence rates in ICUs compared with universal screening and might therefore represent an interesting strategy. The total number of rectal swabs should decrease with targeted screening. The aim of the present study was to assess the impact of targeted screening at ICU admission on 3GC-RE-related HAI incidence in a quasi-experimental setting.

## Material and methods

### Setting, subjects and design

**Figure 1:**
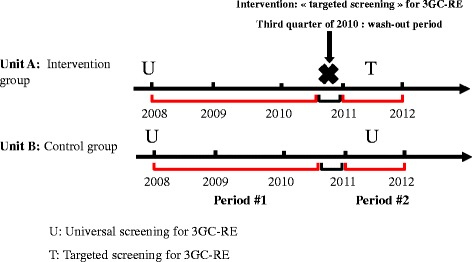
**Design of the quasi-experimental study in two intensive care units of Edouard Herriot Hospital, Lyon, France, 2008 through 2011.** The intervention group was unit A, in which targeted screening for 3GC-RE at patient admission was implemented at the end of 2010. The control group was unit B, in which universal screening at patient admission was performed throughout the study period. Period 1 comprised patients admitted between 2008 and the third quarter of 2010. Period 2 comprised patients admitted in the 2011 calendar year.

We performed a 3-year quasi-experimental study with intervention and control groups to evaluate targeted screening for 3GC-RE at ICU admission. Data were collected prospectively between January 2008 and December 2011 at Edouard Herriot Hospital, an 850-bed university-affiliated hospital in Lyon, France. Two ICUs located in separate buildings were included in the study: Unit A, the intervention group, comprised a polyvalent medical ICU with 15 single rooms; and unit B, the control group, comprised a polyvalent medical-surgical ICU with 12 single rooms. These two ICUs had a similar staff–patient allocation. Data from two different time periods were collected. Figure 1 outlines the study design. Period 1 (pre-test period) included patients admitted between 1 January 2008 and 30 September 2010. During this time period, both units A and B undertook universal screening for 3GC-RE at patient admission. The intervention was the implementation of targeted screening for 3GC-RE at patient admission in the last quarter of 2010 in unit A. After the intervention, the posttest period (period 2) included patients admitted between 1 January 2011 and 31 December 2011. Targeted screening for 3GC-RE at patient admission was implemented in unit A. During this period, unit B maintained universal screening for 3GC-RE at patient admission. The last quarter of 2010 was a wash-out period for targeted screening in unit A. The incidence rates of overall HAIs and 3GC-RE-related HAIs and the total number of rectal swabs between the two time periods were compared to assess the effectiveness of targeted screening.

The two study ICUs have participated in the French National HAI Surveillance Network in Intensive Care Units (Réseau REA-RAISIN) since 1999. The programme is described in detail in the literature [24-26]. All patients hospitalized for ≥48 hours in either unit A or B during the study period (except for the washout period at the end of 2010) were included in the national surveillance network and participated in the study. Data were collected prospectively by the hospital infection control team (nurses and physicians), and feedback was provided annually to ICU physicians and nurses. Furthermore, a team member was regularly present in both units to remind ICU staff of prevention practices. Infection control strategies were similar in both units during the surveillance periods, and written protocols were available. Universal precautions (including regular hand hygiene with alcohol-based solution and wearing gloves and gown during activities likely to generate contact with body fluids) were applied throughout the study. Barrier precautions (that is, rigorous handwashing, strict use of gowns and gloves) were enforced for all 3GC-RE-positive patients, as recommended by the Centers for Disease Control and Prevention [27]. Counselling by the expert infection control team was similar during the surveillance period. No educational programmes were engaged in during the study.

### Definitions of active screening culture and microbiological methods

*Universal screening* for 3GC-RE was defined as rectal swab screening ≤48 hours after patient admission. All patients submitted to contact isolation pending the results. If samples were negative for 3GC-RE, contact isolation was lifted and rectal swabs were repeated weekly until patient discharge. Contact isolation was maintained if samples were 3GC-RE-positive.

*Targeted screening* for 3GC-RE was defined as rectal swab screening ≤48 hours after admission only for patients transferred from another unit or hospital to the ICU (long-term care facilities and nursing homes included). Patients transferred from an emergency department were excluded if they were admitted from home. All screened patients were placed in contact isolation pending the results. If samples were 3GC-RE-negative, contact precautions were stopped and rectal swabs were not repeated weekly. Patients with a history of 3GC-RE asymptomatic carriage or infection in their medical records were directly placed in contact isolation without sampling. In contrast, patients were not screened for 3GC-RE if they were not transferred from another unit or hospital to the ICU.

Rectal swabs were taken to detect 3GC-RE and were plated in 5 mg/L ceftazidime-based Mueller Hinton medium (BD, Pont de Claix, France). The plates were incubated for 18 to 24 hours in aerobic conditions, and all colonies with growth in medium were identified by mass spectrometry (Saramis; bioMérieux, Marcy l’Etoile, France). Carriers were defined as patients from whom 3GC-RE was recovered from screening samples. The results of screening cultures for 3GC-RE were analysed by using the MOLIS laboratory information system (vision4health, Paris, France).

### Definitions of overall HAIs and 3GC-RE-related HAIs

Overall HAIs and 3GC-RE-related HAIs were recorded in accordance with surveillance network definitions, are based on national guidelines of the French Ministry of Health [28], and in accordance with European Centre for Disease Prevention and Control definitions [29]. Three HAI types were included in the REA-RAISIN and then in the surveillance: pneumonia, urinary tract infections and bacteraemia. Only the first HAI occurring >48 hours after patient admission was taken into account. All types of bacteria may have been implicated in overall HAIs. 3GC-RE was defined as *Enterobacteriaceae* resistant to third-generation cephalosporins, regardless of the factor contributing to cephalosporin resistance (that is, extended-spectrum β-lactamase (ESBL) or overproduction of cephalosporinase). 3GC-RE-related HAI was defined by the identification of at least one 3GC-RE in a clinical sample.

Pneumonia was defined according to the following criteria:

1. Two chest X-rays exhibiting lung infiltrates; and
2. At least one of the following clinical signs:
  - Hyperthermia (>38°C) without any other cause and/or
  - Leucopenia (leucocyte count <4,000 white blood cells (WBCs)/mm^3^) or leucocytosis (leucocyte count >12,000 WBCs/mm^3^); and
3. At least one of the following:
  - Onset of purulent secretions or changes in characteristics,
  - Findings suggestive of auscultation and/or
  - Cough, dyspnoea or tachypnoea and
  - Low oxyhaemoglobin saturation or increased pulmonary oxygen consumption; and
4. Diagnostic method:
  - Directed bronchoalveolar lavage (BAL)–positive culture at a threshold of 10^4^ colony forming units (cfu)/ml in BAL or 10^3^ cfu/ml in mini-BAL,
  - Fibre-optic bronchoscopy specimen-positive culture at a threshold of 10^6^ cfu/ml or
  - One of the following alternative methods: positive blood culture without any other sources of infection, positive culture of pleural fluid, pleural or lung abscess with positive culture or histological evidence of pneumonia.

Urinary tract infection was defined by the following criteria:

1. Fever (>38°C), feeling of urinary urgency, frequent urination, dysuria, burning sensation, suprapubic pain in the absence of any other cause, infectious or noninfectious ; and
2. With current catheterization or catheterization in the preceding 7 days: positive urine culture (≥10^5^ cfu/ml) and, at the most, two different microorganisms; and
3. Without catheterization: leucocyturia (≥10^4^ cells/ml) and positive urine culture (≥10^3^ cfu/ml) and, at the most, two different microorganisms.

Bacteraemia was defined as an association of clinical signs and at least one positive blood culture isolate. Two positive blood cultures with the same microorganism were needed for the following microorganisms: coagulase-negative staphylococci, *Bacillus* spp. (except *Bacillus anthracis*), *Corynebacterium* spp*.*, *Propionibacterium* spp*.*, *Micrococcus* spp*.* or other saprophytic or commensal microorganisms with comparable pathogenic potential. Blood cultures should be collected from different sites and at different times (maximum of 48 hours is usual).

### Statistical analysis

The continuous variables evaluated were as follows: length of ICU stay (days), age (years), Simplified Acute Physiology Score II (SAPS II), length of mechanical ventilation and urinary catheterization (days). These variables were analysed as mean and standard deviation (SD). Age and SAPS II were further grouped and coded for analysis by age group (0 to 44, 45 to 59, 60 to 74 and 75+ years) and SAPS II (scores: 0 to 29, 30 to 39, 40 to 49 and 50+). The categorical variables analysed included sex, antibiotics received at admission, patient origin, diagnostic category at admission, trauma patients (with or without surgical intervention), immunodeficiency, invasive devices and in-hospital death. Analyses were conducted with Stata 11.0 software (StataCorp, College Station, TX, USA).

Descriptive analysis of the study population was undertaken. It was supplemented by analysis of the unit population over time. Analyses for trends in the incidence of overall HAIs and 3GC-RE-related HAIs were performed. Qualitative variables are reported as number of individuals and percentage, with the χ^2^ or Fisher’s exact test used as appropriate. Quantitative variables are reported as mean and SD and were analysed by *t*-test. The attack rate was defined as the number of overall HAIs or 3GC-RE-related HAIs per 100 patients, and incidence was defined the number of HAIs per 1,000 patient-days at risk. The evolution of the number of rectal swabs and the number of patients sampled at admission in the two units before and after the intervention were modelled by a Poisson regression that included the number of samples, the time trend (in months) and the period (1 or 2).

The Poisson regression modelled the effect of time trends on 3GC-RE-related HAI incidence rates. In these regression analyses, HAI rates were dependent variables, and time trends (in quarters) and period were independent variables. The potential confounders tested were sex, age category, SAPS II, diagnostic category at admission, patient origin, diagnostic category, central venous catheterization, mechanical invasive ventilation, urinary catheterization and antibiotics received at admission. Time trend and period were forced into the model for multivariate analysis. Other variables were introduced in multivariate analysis if *P* < 0.15 in univariate analysis. The final model contained the two independent variables and all the potential confounders. *P* < 0.05 was considered as statistically significant.

### Ethics statement

The study did not require ethics committee approval, because it was based on an observational surveillance database approved under French national regulations (Comité National Informatique et Liberté). Written consent was not obtained from patients, because we carried out a retrospective investigation, the data were analysed anonymously and patient care was not affected.

## Results

### Patient characteristics

Over the 3-year study period, 2,915 patients in both ICUs were hospitalized for ≥48 hours. Among them, 189 (6.5%) were hospitalized in the last quarter of 2010 and were therefore excluded. One hundred sixty-eight patients (6.2%) had one or more missing values and were also excluded; of these, 99 (5.6%) were hospitalized in unit A and 69 (7.1%) in unit B (*P* = 0.1). Ultimately, 2,558 patients, accounting for 25,769 patient-days, were included in the study: 1,756 (68.6%) patients accounted for 15,149 patient-days in unit A, and 802 (31.4%) patients accounted for 10,620 patient-days in unit B.

In the study population, 1,604 (62.7%) were male, 1,631 (63.8%) received antibiotics at admission, 1,115 (43.6%) came from home and 775 (30.1%) were immunosuppressed. The mean age was 61.1 ± 16.7 years (median age, 63 years; interquartile range, 51 to 74), and the mean SAPS II was 47.6 ± 19.7. The mean length of hospital stay was 10.1 ± 17.0 days; it was 8.9 ± 17.8 days for invasive mechanical ventilation and 9.2 ± 14.6 days for urinary catheterization.

Table 1 describes patient characteristics by unit and time period. Some characteristics differed between periods 1 and 2. In unit A, the proportions of patients differed between the two periods as follows: trauma patients, intubated patients and patients with urinary catheters; patient origin; proportion of antibiotic therapy at admission; and mean duration of stay, of intubation and of urinary catheterization. The proportions differed over time in unit B in the following groups: the proportions of trauma, intubated and catheterized patients; the proportions of those on antibiotic therapy at admission and of immunodepressed patients; patient origin and diagnostic category; and mean age and SAPS II.

**Table 1:** **Study population and hospital-acquired infection incidence rates by unit and time period** ^**a**^

**Variables**		**Unit A**			**Unit B**		
		**Period 1** **(2008 to 2010)** ^**c**^ **,** ***n*** **= 1,110**	**Period 2** **(2011)** ^**c**^ **,** ***n*** **= 552**	***P*** **-value**	**Period 1** **(2008 to 2010)** ^**b**^ **,** ***n*** **= 646**	**Period 2** **(2011)** ^**b**^ **,** ***n*** **= 250**	***P*** **-value**
Categorical variables, *n* (%)							
	Male sex	669 (60.3)	348 (63.0)	0.28	430 (66.6)	157 (62.8)	0.29
	Antibiotics^d^	759 (68.4)	436 (79.0)	<0.001	288 (44.6)	148 (59.2)	<0.001
	Trauma patients	44 (4.0)	8 (1.5)	0.006	137 (21.2)	15 (6.0)	<0.001
Diagnostic category^d^				0.14			0.004
	Medical ICU	1,030 (92.8)	522 (94.6)		248 (38.4)	121 (48.4)	
	Emergency surgical ICU	50 (4.5)	14 (2.5)		181 (28.0)	46 (18.4)	
	Scheduled surgical ICU	30 (2.7)	16 (2.9)		217 (33.6)	83 (33.2)	
Patient origin^d^				<0.001			0.005
	Home or nursing home	473 (42.6)	437 (79.2)		140 (21.7)	65 (26.0)	
	Short-stay unit	559 (50.4)	103 (18.7)		404 (62.5)	146 (58.4)	
	Long-stay unit	29 (2.6)	7 (1.3)		24 (3.7)	0 (0.0)	
	ICU	49 (4.4)	5 (0.9)		78 (12.1)	39 (15.6)	
	Immunodeficiency^d^	243 (21.9)	102 (18,5)	0.16	329 (51.0)	101 (40.4)	0.014
	Invasive mechanical ventilation	800 (72.1)	369 (66.9)	0.03	442 (68.4)	131 (52.4)	<0.001
	Central venous catheterization	737 (66.4)	383 (69.4)	0.22	518 (80.2)	220 (88.0)	0.006
	Urinary catheterization	1,027 (92.5)	494 (89.5)	0.04	557 (86.2)	217 (86.8)	0.82
	Died in-hospital	253 (22.8)	109 (19.8)	0.16	93 (14.4)	37 (14.8)	0.88
Continuous variable, mean (SD)							
	Length of ICU stay, days	10.1 (15.4)	7.1 (8.5)	<0.001	12.1 (22.8)	11.1 (19.0)	0.54
	Age, years^d^	61.2 (16.9)	62.7 (16.9)	0.10	59.1 (16.0)	61.8 (16.6)	0.03
	SAPS II^d^	51.7 (19.1)	51.6 (18.3)	0.92	41.2 (20.0)	37.7 (16.3)	0.014
	Length of invasive mechanical ventilation, days	8.7 (15.1)	5.7 (7.8)	<0.001	11.4 (25.7)	11.0 (20.0)	0.85
	Length of urinary catheterization, days	9.7 (14.1)	6.2 (6.5)	<0.001	10.6 (19.2)	9.6 (15.8)	0.49
Incidence of overall HAIs							
	Number of HAIs	149	50		116	38	
	Attack rate^e^	13.4	9.1	0.01	18.0	15.2	0.33
	Incidence of overall HAIs^f^ (95% CI)	18.0 (15.3 to 21.2)	15.2 (11.3 to 20.0)	0.29	23.5 (19.4 to 28.2)	21.8 (15.5 to 30.0)	0.71
Incidence of 3GC-RE-related HAIs							
	Number of 3GC-RE-related HAIs	56	5		20	5	
	Attack rate^e^	5.1	0.9	<0.001	3.1	2.0	0.37
	Incidence of 3GC-RE-related HAIs^f^ (95% CI)	5.4 (4.1 to 7.0)	1.3 (0.4 to 3.0)	<0.001	2.8 (1.7 to 4.3)	1.9 (0.6 to 4.5)	0.48

^a^CI, Confidence interval; 3GC-RE-related HAIs, Hospital-acquired infections (pneumonia, urinary tract infections and bacteraemia) with identification of third-generation cephalosporin-resistant *Enterobacteriaceae* in at least one clinical sample; ICU, Intensive care unit; Overall HAIs, Overall hospital-acquired infections (pneumonia, urinary tract infections and bacteraemia); SAPS II, Simplified Acute Physiology Score II; SD, Standard deviation. ^b^Universal screening. ^c^Targeted screening. ^d^At admission. ^e^Per 100 patients. ^f^Per 1,000 patient-days.

Overall, 149 patients (5.8%) developed *Enterobacteriaceae*-related HAIs. For 23 of these patients, antibiotic resistance of the identified bacteria was not tested (*P* = 0.06 between period 1 and 2). 3GC-REs were identified in 86 (3.4%) of 149 infected patients: 61 (3.7%) in unit A and 25 (2.8%) in unit B. Among these patients, 50 (58.1%) were admitted from another hospital unit. Ninety-three 3GC-REs were isolated from clinical samples, 13 (14.0%) of them were both 3GC- and carbapenem-resistant. The most common bacteria isolated in 3GC-RE-related HAIs were *Klebsiella* spp. (*n* = 24, 37.0%) in unit A and *Escherichia coli* (n = 9, 32.1%) in unit B. Attack rates of first 3GC-RE-related HAIs per 100 admissions were comparable in units A and B (*P* = 0.24). Nevertheless, the incidence rate of 3GC-RE-related HAIs per 1,000 patient-days was statistically higher in unit A (4.3; 95% CI, 3.3 to 5.5) than in unit B (2.5; 95% CI, 1.7 to 3.7) (*P* = 0.03).

### Effect of targeted screening for 3GC-RE on HAI incidence

**Figure 2:**
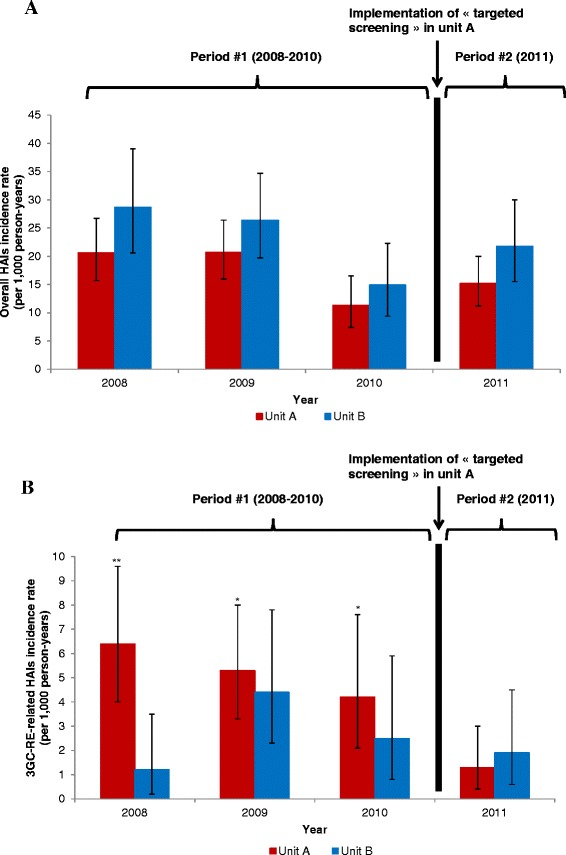
**Trends of overall and third-generation, cephalosporin-resistant** ***Enterobacteriaceae*** **–related hospital-acquired infections by period and by unit. (A)** Trends of overall hospital acquired infections (HAIs) by period and unit are depicted. *Note*: For each unit, the incidence rate per year (2008, 2009 and 2010) was compared with the rate in 2011. The intervention group was unit A with targeted screening for 3GC-RE at patient admission at the end of 2010. The control group was unit B with universal screening at patient admission throughout the study. Period 1 comprised patients admitted between 2008 and the third quarter of 2010. Period 2 comprised patients admitted in the 2011 calendar year. In both units, the overall HAI incidence rate remained stable over time. **(B)** Third-generation, cephalosporin-resistant *Enterobacteriaceae* (3GC-RE)–related HAI trends by period and unit are shown. *P < 0.05. **P < 0.001. For each unit, the incidence rate per year (2008, 2009 and 2010) was compared with the rate in 2011. The intervention group was unit A with targeted screening for 3GC-RE at the end of 2010. The control group was unit B with universal screening throughout the study. Period 1 comprised patients admitted between 2008 and the third quarter of 2010. Period 2 comprised patients admitted in 2011. In unit A, the 3GC-RE-related HAI incidence rate decreased during period 2 compared with each year of period 1. In unit B, no significant decrease was observed between periods 1 and 2.

Table 1 reports attack and incidence rates of overall HAIs and 3GC-RE-related HAIs. In both units, incidence rates of overall HAIs remained stable during period 1 compared with period 2 (*P* = 0.29 in unit A, *P* = 0.71 in unit B). Incidence rates of overall HAIs by year and by unit are shown in Figure 2A. In unit A, the intervention group, the 3GC-RE-related HAI incidence rate decreased from 5.4 (95% CI, 4.1 to 7.0) during period 1 to 1.3 (95% CI, 0.5 to 2.9) during period 2 (*P* < 0.001). In unit B, the control group, the 3GC-RE-related HAI incidence rate remained stable during period 2 compared with period 1 (*P* = 0.48). 3GC-RE-related HAI incidence rates by year and by unit are shown in Figure 2B.

The results of multivariate Poisson regression analysis are presented in Table 2. Age and trauma patients were found to have a significant effect (*P* < 0.05) and were retained in the final multivariate model. The multivariate model was forced with temporal trend and period. Adjusted incidence decreased after implementation of targeted screening in unit A (incidence rate ratio (IRR) = 0.28; 95% CI, 0.09 to 0.88; *P* = 0.03). No decrease was shown in unit B between periods 1 and 2 (*P* = 0.40).

**Table 2:** **Incidence rate ratios of third-generation, cephalosporin-resistant** ***Enterobacteriaceae–*** **related hospital-acquired infections with multivariate poisson regression analysis** ^**a**^

**Variables**			**Intervention group: unit A**		**Control group: unit B**	
			**Adjusted IRR of 3GC-RE HAIs** **(95% CI)** ^**b**^	***P*** **-value**	**Adjusted IRR of 3GC-RE HAIs** **(95% CI)** ^**b**^	***P*** **-value**
Bivariate model^c^						
	Time trend, per quarter^d^		0.97 (0.89 to 1.06)	0.50	1.05 (0.91 to 1.21)	0.52
	Period					
		Before targeted screening^e^	1		1	
		After targeted screening^f^	0.30 (0.098 to 0.94)	0.04	0.50 (0.12 to 2.01)	0.32
Multivariate model^g^						
	Time trend, per quarter^d^		0.99 (0.91 to 1.08)	0.85	1.01 (0.88 to 1.17)	0.18
	Period					
		Before targeted screening^e^	1		1	
		After targeted screening^f^	0.28 (0.090 to 0.88)	0.03	0.54 (0.13 to 2.25)	0.40

^a^3GC-RE, Third-generation cephalosporin–resistant *Enterobacteriaceae*; HAI, Hospital-acquired infection; IRR: Incidence rate ratio. ^b^After multivariate Poisson regression. ^c^Time trend (per quarter) and period were forced into the model. ^d^3-month interval. . ^e^Period 1 (2008 to 2010). ^f^Period 2 (2011). ^g^Adjusted for age and trauma patients. Time trend (per quarter) and period were forced in the model.

### Effect of targeted screening on the number of rectal swabs sampled

In total, 2,899 rectal swabs were collected in both ICUs: 1,112 (38.5%) in unit A and 1,787 (61.6%) in unit B. A total of 1,589 samples were collected at admission. One hundred eighteen (7.4%) of screened patients tested positive for 3GC-RE at admission during the study period: 64 (8.0%) in unit A and 54 (6.4%) in unit B (*P* = 0.4). Table 3 describes the different 3GC-REs identified from rectal swabs at patient admission. The most common bacteria isolated at patient admission were *Klebsiella* spp. (*n* = 26 bacteria, 40.6%) in unit A and *Escherichia coli* and *Enterobacter* spp. (*n* = 16 bacteria, 29.6%) in unit B. Among the 1,471 (92.6%) patients with negative 3GC-RE cultures at admission, 85 (5.8%) acquired 3GC-RE colonization: 32 (4.3%) in unit A and 53 (7.2%) in unit B (*P* = 0.02). Among the 118 (7.4%) patients colonized with 3GC-RE at admission, 22 (18.6%) of colonized patients subsequently had negative cultures and 88 (74.6%) remained colonized. Data about the clinical evolution of infection during the stay were found for 99 patients (84% of colonized patients at admission). Fifty-seven (57.6%) patients were admitted from another hospitalization unit or nursing home. Eleven (11.1%) developed an infection due to 3GC-RE during the stay. Among these 11 patients, 8 (72.7%) were admitted from another hospitalization unit.

**Table 3:** **Description of third-generation, cephalosporin-resistant** ***Enterobacteriaceae*** **isolated from rectal swabs at patient admission**

**Bacteria,** ***n*** **(%)**	**Unit A,** ***n*** **= 64**	**Unit B,** ***n*** **= 54**	***P*** **-value**	**All units,** ***n*** **= 118**
*Klebsiella pneumoniae*	26 (40.6)	14 (25.9)	0.09	40 (33.9)
*Enterobacter* spp.	19 (29.7)	16 (29.6)	0.9	35 (29.7)
*Escherichia coli*	13 (20.3)	16 (29.6)	0.3	29 (24.6)
*Citrobacter* spp.	3 (4.7)	4 (7.4)	0.6	7 (5.9)
*Morganella morganii*	2 (3.1)	1 (1.9)	0.7	3 (2.5)
*Pantoea* spp.	0 (0.0)	2 (3.7)	0.2	2 (1.7)
*Serratia marcescens*	1 (1.6)	0 (0.0)	0.5	1 (0.8)
*Hafnia alvei*	0 (0.0)	1 (1.9)	0.5	1 (0.8)

**Figure 3:**
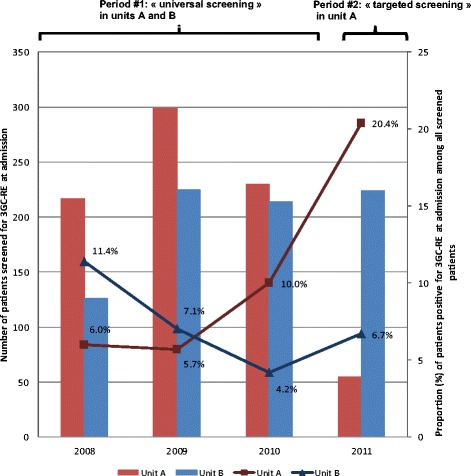
**Number of patients screened for third-generation, cephalosporin-resistant** ***Enterobacteriaceae*** **at admission and proportion of patients positive for third-generation, cephalosporin-resistant** ***Enterobacteriaceae*** **.** Red columns represent the number of patients screened for third-generation, cephalosporin-resistant *Enterobacteriaceae* (3GC-RE) in unit A. Blue columns represent the number of patients screened for 3GC-RE in unit B. The lines represent the proportion of patients positive for 3GC-RE at admission among all screened patients at admission for each unit. In unit A, the intervention unit, the number of patients screened at admission was divided by 5 between period 1 (universal screening, 2008 to 2010) and period 2 (targeted screening, 2011). The proportion of patients positive for 3GC-RE at admission increased between periods 1 and 2 (*P* = 0.004). No changes were observed in unit B, the control unit, between periods 1 and period 2 (*P* = 0.98).

The total number of samples decreased by 84.7% (95% CI, 80.0% to 88.2%; *P* < 0.001) in unit A between periods 1 and 2. No decrease was observed in unit B during period 2 (*P* = 0.44). The number of patients screened for 3GC-RE at admission and proportions of patients with 3GC-RE carriage detected at admission are shown in Figure 3 by year and by time period. The number of patients screened for 3GC-RE at admission decreased by 80.0% (95% CI, 73.7% to 84.9%; *P* < 0.001) between periods 1 and 2 in unit A. Conversely, no decline was apparent in unit B between periods 1 and 2 (*P* = 0.42). In unit A, the proportion of patients with 3GC-RE carriage detected at admission among screened patients was significantly higher during period 2 (20.4 per 100 screened patients) compared with period 1 (7.1 per 100 screened patients) (*P* = 0.003). In unit B, no difference was observed over time (*P* = 0.99).

## Discussion

Identification of 3GC-RE carriers by rectal screening at admission seems to be an important step in the prevention of transmission and outbreaks in ICUs; unlike methicillin-resistant *Staphylococcus aureus* (MRSA) and vancomycin-resistant *Enterococcus* [30,31], however, little is known about its effectiveness [12]. The objective of this study was to evaluate the impact of targeted screening compared with universal screening at admission on incidence rates of 3GC-RE-related HAIs in ICUs.

The main finding of this study is the absence of an increase of the incidence density of 3GC-RE-related HAIs (per 1,000 patient-days) during period 2 (‘targeted screening’) compared with period 1 (‘universal screening’) in unit A, even in multivariate models. A strength of the study is that HAI surveillance is included in the REA-RAISIN. Thus, HAI definitions did not change over the study period, and data collection followed a standardized protocol, limiting collection bias. Moreover, the study was carried out in a nonoutbreak setting. Infection control measures were the same over time (that is, standard precautions and contact isolation of 3GC-RE-positive patients).

In addition, the results of our study show that the number of patients screened for 3GC-RE at admission decreased in unit A during period 2. Among patients who tested positive for 3GC-RE carriage at admission, less than 15% developed an infection due to 3GC-RE. Conversely, nearly three-fourths of patients who tested positive for 3GC-RE at admission and became infected by 3GC-RE during their hospitalization were transferred from another hospitalization unit. These results reveal that targeted screening at admission decreased the workload of ICU staff and permitted them to target those patients at greatest risk for 3GC-RE infection among all patients colonized by C3G-RE at admission.

To avoid patient-to-patient transmission, it may be important to detect patients colonized by 3GC-RE at admission to ICUs [17]. For several reasons, universal screening may not be an optimal ICU strategy. First, the proportion of patient-to-patient transmissions remains somewhat unknown and seems to vary from species to species [32,33]. In some studies, researchers have tried to evaluate patient-to-patient transmission by analysing the genetic similarity of isolates, but the results appear to be discrepant [34]. Moreover, *Escherichia coli* is increasingly implicated in infections [35], and some evidence of low patient-to-patient transmission [32] may challenge interest in universal screening. Universal screening does not appear to be cost-effective for ESBL-E eradication [16,36]. It was reported to be more than CAD $1 million in a 2002 study [15]. Thus, targeting patients at risk of colonization at ICU admission may be worth further assessment as an alternative to universal screening. There is in choosing criteria for targeted screening at ICU admission. Transfer from another hospital unit is known to be a risk factor for 3GC-RE carriage [21]. This criterion was chosen in unit A in the present study for its simplicity because transferred patients are easy to identify. However, other risk factors might be spotlighted according to local 3GC-RE epidemiology. Targeting patients with a high risk of nosocomial infection and in whom adequate antibiotic treatment is warranted (for example, patients with severe trauma, major surgery) is also worth considering [18].

SDD could be viewed as a competing strategy. Indeed, universal decolonization was found to be more effective than targeted screening and isolation in reducing rates of MRSA clinical isolates [37], but its efficacy is not fully known in the case of 3GC-RE. Some researchers have reported positive results with decolonization of carriers in ICU [38], even if the emergence of resistance was a particular concern [39].

A 2005 to 2009 Canadian study suggested that the higher the proportion of screened patients, the lower the 3GC-RE-related HAI incidence rate [40], which could be explained by a higher number of patients in contact isolation and decreased patient-to-patient transmission. This assumption runs counter to our results, which show no increase of the 3GC-RE-related HAI incidence rate with a lower proportion of screened patients. However, various individual and facility factors influencing 3GC-RE acquisition might explain the difference [34]. Compliance with barrier precautions, as well as other noncollected data (such as previous antibiotic therapies), was not evaluated in our study. Thus, even if our results may not be extended, targeted screening for 3GC-RE at ICU admission seemed to be as effective as universal screening in our study.

Our investigation has some potential limitations. First, its quasi-experimental design is limited by a lack of randomisation. Randomisation, which requires human and financial resources, is indeed tricky to implement in health care settings. The unicentric design does not permit extension of the data. The key strength of our study is the prospective nature of surveillance based on a national, standardized protocol. Moreover, it follows the guidelines for quasi-experimental study design and statistical analysis recommended in the literature [41-43], that is, adjustment on temporal trends and confounding factors. Inclusion of a control group improves the study’s internal validity.

Given the nature of our study, based on a surveillance network protocol, we did not collect all confounding factors described in the literature. Covariables may be incomplete or unsuitable. For instance, the nature and duration of antibiotic administration at admission were not specified. Some comorbidities (such as diabetes mellitus) were not recorded and could have been confounding factors. Some patients’ characteristics changed between periods 1 and 2 in both ICUs, especially length of ICU stay, showing that patients hospitalized in both ICUs changed over time. Nevertheless, the absence of an increase in the 3GC-RE-related HAI incidence rate was still apparent after multivariate analysis, which permitted adjustment for potential confounders. The decreasing incidence may be due to an unidentified change of a noncollected factor over time in one or the other unit. Whereas the 3GC-RE-related HAI incidence rate was high during period 1, it might have decreased during period 2, even in the absence of intervention. Nevertheless, we can hypothesize that the decreased workload associated with targeted screening led to increased infection control compliance regarding 3GC-RE-colonized patients.

3GC-REs are due mainly to ESBL on one hand and overproduction of cephalosporinase on the other hand. Whereas ESBLs are carried by mobile elements, cephalosporinases have mainly chromosomal support. However, plasmid-mediated cephalosporinases have been described in the literature. For example, in a French multicentre ICU study of the prevalence and molecular epidemiology of resistance to 3GCs for *E. coli*, 41 3GC-R *E. coli* were isolated, of which 19 (46.3%) strains were ESBL-producing *E.coli* and 18 (43.9%) were the AmpC phenotype. Among these 18 strains, 5 (27.8%) of the plasmids carried AmpC enzyme [44]. Regarding the cost and workload required for ESBL diagnoses, notably in bacteria among ESBL that could be masked by cephalosporinase, and the emergence of plasmid mediated cephalosporinases, the lack of distinction between ESBL-E and 3GC-RE is a minor limitation of the study. Between 2008 and 2011, the proportions of ESBLs in clinical samples were 84.2% in unit A (144 of 171 clinical samples) and 70.8% in unit B (68 of 96 clinical samples).

## Conclusions

Our study results indicate that the implementation of a relatively simple procedure, consisting of targeted screening for 3GC-RE at ICU admission for transferred patients, does not increase the 3GC-RE-related HAI incidence rate. Universal screening for 3GC-RE requires human and financial resources [36], and targeted screening may be worth further assessment as an alternative to universal screening. However, it remains difficult to recommend such a strategy before robust evidence of proportions of patient-to-patient transmission acquired in multicentre studies has been provided. In addition, controlled and cost-effectiveness studies are warranted to confirm potential interest in targeted screening for 3GC-RE at admission.

## Key messages

- The efficacy of universal screening to detect 3GC-RE carriers at ICU admission on the 3GC-RE HAI incidence rates is imperfectly known and seems not to be cost-effective, particularly if patient-to-patient transmission or prevalence at admission is low.
- The aim of the study was to evaluate the impact of targeted screening for 3GC-RE at ICU admission on the 3GC-RE HAI incidence rate and compare it to universal screening.
- A quasi-experimental study with intervention and control groups showed that the implementation of targeted screening for 3GC-RE at ICU admission for patients transferred from another unit or hospital does not involve an increase of 3GC-RE HAI incidence over time.
- An 85% decrease in the total number of rectal samples was observed.
- Targeted screening may be worthy of further assessment as an alternative to universal screening.

## Abbreviations

ASC: Active screening culture
BAL: Bronchoalveolar lavage
CI: Confidence interval
ESBL: Extended-spectrum β-lactamase
ESBL-E: Extended-spectrum β-lactamase-producing *Enterobacteriaceae*
HAI: Hospital-acquired infection
ICU: Intensive care unit
IRR: Incidence rate ratio
MRSA: Methicillin-resistant *Staphylococcus aureus*
SAPS II: Simplified Acute Physiology Score II
SD: Standard deviation
SDD: Selective digestive decontamination
3GC-RE: Third-generation cephalosporin-resistant *Enterobacteriaceae*
